# HIV-1 Nef Sequence and Functional Compartmentalization in the Gut Is Not Due to Differential Cytotoxic T Lymphocyte Selective Pressure

**DOI:** 10.1371/journal.pone.0075620

**Published:** 2013-09-13

**Authors:** Martha J. Lewis, Patricia Frohnen, F. Javier Ibarrondo, Diane Reed, Varun Iyer, Hwee L. Ng, Julie Elliott, Otto O. Yang, Peter Anton

**Affiliations:** 1 Department of Medicine, Division of Infectious Diseases, David Geffen School of Medicine at UCLA, Los Angeles, California, United States of America; 2 Division of Digestive Diseases, David Geffen School of Medicine at UCLA, Los Angeles, California, United States of America; 3 UCLA AIDS Institute, David Geffen School of Medicine at UCLA, Los Angeles, California, United States of America; National Institute of Infectious Diseases, Japan

## Abstract

The gut is the largest lymphoid organ in the body and a site of active HIV-1 replication and immune surveillance. The gut is a reservoir of persistent infection in some individuals with fully suppressed plasma viremia on combination antiretroviral therapy (cART) although the cause of this persistence is unknown. The HIV-1 accessory protein Nef contributes to persistence through multiple functions including immune evasion and increasing infectivity. Previous studies showed that Nef’s function is shaped by cytotoxic T lymphocyte (CTL) responses and that there are distinct populations of Nef within tissue compartments. We asked whether Nef’s sequence and/or function are compartmentalized in the gut and how compartmentalization relates to local CTL immune responses. Primary *nef* quasispecies from paired plasma and sigmoid colon biopsies from chronically infected subjects not on therapy were sequenced and cloned into Env^−^ Vpu^−^ pseudotyped reporter viruses. CTL responses were mapped by IFN-γ ELISpot using expanded CD8+ cells from blood and gut with pools of overlapping peptides covering the entire HIV proteome. CD4 and MHC Class I Nef-mediated downregulation was measured by flow cytometry. Multiple tests indicated compartmentalization of *nef* sequences in 5 of 8 subjects. There was also compartmentalization of function with MHC Class I downregulation relatively well preserved, but significant loss of CD4 downregulation specifically by gut quasispecies in 5 of 7 subjects. There was no compartmentalization of CTL responses in 6 of 8 subjects, and the selective pressure on quasispecies correlated with the magnitude CTL response regardless of location. These results demonstrate that Nef adapts via diverse pathways to local selective pressures within gut mucosa, which may be predominated by factors other than CTL responses such as target cell availability. The finding of a functionally distinct population within gut mucosa offers some insight into how HIV-1 may persist in the gut despite fully suppressed plasma viremia on cART.

## Introduction

Nef is a multifunctional accessory protein encoded by HIV and SIV that plays a key role in pathogenesis. Although Nef is dispensable for *in vitro* replication, *in vivo* data from both humans and animal models support Nef’s important role in causing disease [1]. Macaques infected with an otherwise pathogenic strain of SIV with *nef* deleted tend to have significantly attenuated disease and protection from subsequent challenge with wild type virus [2], [3]. Similarly, slower disease progression has been noted in a cohort of humans infected by blood transfusions from a single HIV-1-infected donor whose virus contained a defect in *nef* [4], [5], [6]. Nef mediates its pathogenic effects via binding to a wide variety of cellular factors resulting in increased infectivity, increased replication and evasion of host immune responses [7]. Elimination of some or all of these functions leads to an attenuated infection [8]. Among the best-understood functions of Nef are downregulation of MHC Class I [9], [10] and CD4 [11] molecules from the surface of infected cells. Nef’s downregulation of CD4 prevents its binding to Env on nascent virions thereby enhancing viron release [12]. MHC Class I downregulation has been shown to render infected cells less visible to circulating CD8+ cytotoxic T lymphocytes (CTL) and thereby confer resistance of infected cells to CTL killing [9], [13].

Previous studies have shown that Nef’s function is shaped by cytotoxic T lymphocyte (CTL) responses and varies according to stage of disease. It has been observed that Nef-mediated CD4 and MHC Class I downregulation is preserved across transmission [14] but is diminished or absent by end-stage AIDS [15]. In a cohort of chronically infected subjects it was observed that circulating quasispecies contained mixtures of two distinct populations of virus, one that fully downregulated MHC Class I and the other with no ability to downregulate MHC Class I. Further, the proportion of functional quasispecies correlated with the breadth of the CTL response in these subjects, increasing in response to increased immune pressure demonstrating Nef’s adaptation to the immune milieu of the host [16]. The origin of the functionally distinct quasispecies subsets in peripheral blood is unclear. One possibility is that the different populations are emerging from distinct compartments of viral replication. Local factors such as CTL responses and target cell availability as well as migration rates between tissues and blood all contribute to the degree of differential evolution of HIV in different locations. The balance of local selective pressures and migration rates between tissue compartments may be reflected in the relative proportions of different subsets observed in peripheral blood.

Although HIV-1 sequences are most commonly isolated from peripheral blood, there is evidence that HIV-1 may be evolving differently in tissues. Distinct *env* sequence populations have been observed in the genito-urinary tract in both men and women [17], [18], [19]. Similarly, studies of HIV-1 *env* sequences from different sites within the central nervous system (CNS) demonstrated a clear separation between HIV-1 in the blood and in the brain [20], [21]. Studies specifically of *nef* sequences isolated from the CNS have shown both compartmentalization as well as evidence of adaptation to local selective pressures [22], [23]. An analysis of the function of *nef* alleles in the CNS versus plasma demonstrated the Nef function was relatively well preserved in both compartments [24]. It has also been reported that *nef* quasispecies are compartmentalized in gut tissues. Van Marle et al. observed phylogenetically distinct *nef* populations at several different locations within the gastrointestinal track [25]. However, there is currently no information regarding Nef’s function or adaptation to local selective pressures within the gut.

The gut is now widely recognized as an important site of disease pathogenesis. Gut-associated lymphoid tissue (GALT) is a principal site for HIV-1 replication during the first few weeks of acute infection resulting in the rapid depletion of gut lymphocytes [26], [27]. There is increasing appreciation of the fact that local mucosal responses are important for control of viral replication both in natural infection and vaccination [28], [29], [30]. There is also evidence to suggest that the gut remains a reservoir of replication despite antiretroviral therapy [31], [32] and is key contributor to the persistent immune activation seen in chronic HIV infection [33]. The virus-host interactions within the gut likely have a significant impact on the course of disease, and studies utilizing only peripheral blood may provide an incomplete picture of disease pathogenesis in vivo. Given the importance of the gut as a reservoir and Nef’s key role in viral persistence through its multiple functions we sought to determine whether Nef’s sequence and/or function are compartmentalized within the gut and how compartmentalization relates to local CTL immune responses.

## Results

### Study Subjects

Eight chronically infected HIV-positive subjects were consented to give blood and undergo sigmoidoscopy with tissue biopsy on two study visits 2 weeks apart (Table 1). These subjects were not on any antiretroviral therapy in the proceeding 12 months, as treatment has been shown to diminish CTL responses. Subjects were all seropositive for at least 24 months with a range from 24 to greater than 120 months. All subjects were viremic in the range of 4-5log copies/mL of plasma, and their absolute CD4+ T lymphocyte count from peripheral blood ranged from 41–738 cells/mL of whole blood.

**Table 1:** Study subject information.

*Subject number*	*Viral load (RNA copies/mL)*	*Log Viral load*	*Absolute CD4+ T cell count/mL*	*Months post-seroconversion*
160	98825	4.99	41	82
542	45399	4.66	336	24
608	49713	4.70	738	>120
648	63251	4.80	281	34
650	67380	4.83	245	>120
674	16800	4.23	423	118
677	338202	5.53	222	>120
712	56038	4.75	585	>120

### Nef quasispecies sequences are compartmentalized in the gut

**Figure 1:**
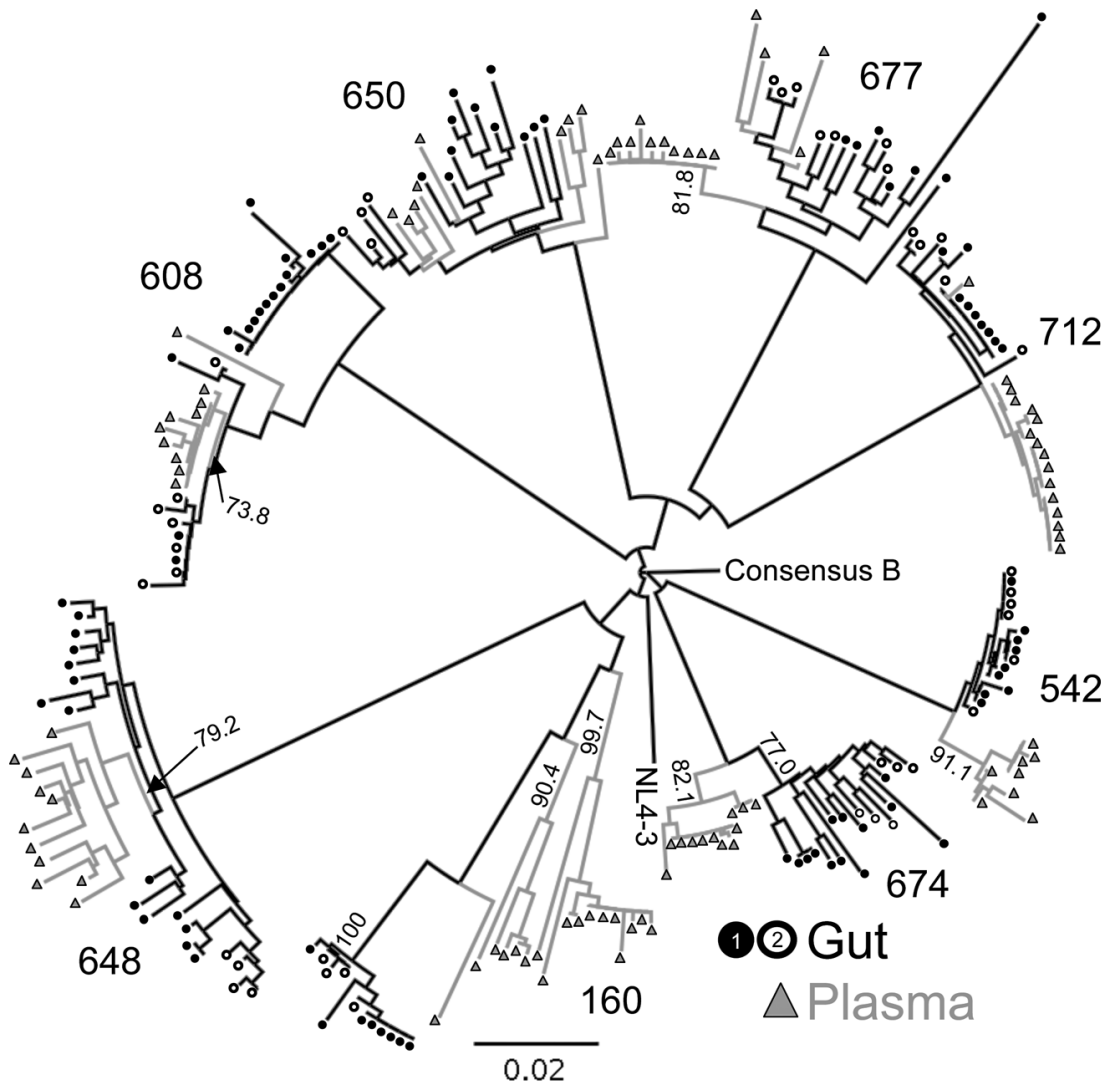
Neighbor-joining phylogenetic tree of plasma and gut-derived *nef* quasispecies. 234 full-length *nef* sequences were aligned with consensus B and NL4-3. A neighbor-joining tree was constructed, rooted on consensus B, and evaluated with 1000 bootstrap replicates. Clusters of sequences from each subject all had 100% bootstrap support and other significant bootstrap values >70% within each subject cluster are indicated on the tree. Sequences from plasma are indicated with grey triangles; gut, black circles. Filled circles are sequences from biopsy #1 and open circles, from biopsy #2.

Multiple full-length individual *nef* clones were sequenced from viral RNA in plasma and from total RNA extracted from the tissue biopsy (N = 234 sequences, median 14.5 sequences from each compartment with a range of 9–23). Sequences were obtained from different biopsies taken 2 weeks apart to control for possible false-positive compartmentalization due to sampling bias caused by the small size of the gut biopsy. There was no evidence of G to A hypermutation in any of the sequences as determined by HyperMut 2.0. Greater than 99% (232/234) of *nef* sequences were intact without missense or nonsense mutations, however, it is not known whether the rest of the genome was fully intact. Neighbor-joining (NJ) (Figure 1) and Maximum Likelihood (ML) (Figure S1) phylogenetic trees were constructed and had very similar topology. The NJ tree was evaluated with 1000 bootstrap replicates. Sequences from each subject formed independent clusters with 100% bootstrap support. Additional independent clusters supported by bootstrap values >70% containing either only gut or plasma isolates were observed in 5 of the 8 subjects (Figure 1 and Table 2).

**Table 2:** Evidence of Compartmentalization.

*Subject number*	*Bootstrap Support* a	*Simmons AI^b^*	*SM migrations^c^*
160	Gut 100%	0.017	1:plasma>gut
542	Plasma 91.1%	0.015	1:plasma>gut
608	Plasma 73.8%	0.17	1:gut>plasma
648	Plasma 79.2%	0.006	1:gut>plasma
650	NS	0.23	Multiple
674	Plasma 77%, Gut 82.1%	0.016	1:plasma>gut
677	NS	0.22	Multiple
712	NS	0.23	Multiple

a Samples of just plasma or just gut isolates having >70% of 1000 bootstrap replicates supporting an independent cluster, NS  =  not significant.

b Association Index. values <0.1 are highly significant for compartmentalization.

c Slatkin-Maddison migration test. “1” indicates a single migration event consistent with compartmentalization, text following indicates the direction of the migration event between plasma and gut. “multiple” indicates multiple migrations or free exchange of sequences between plasma and gut consistent with lack of compartmentalization.

Although the strong bootstrap support for independent clusters of gut or plasma sequences is good evidence of compartmentalization, it has been shown that different methods to assess compartmentalization are subject to different confounding effects and levels of sensitivity sometimes yielding different results [34]. Therefore, two additional tests for compartmentalization, the Slatkin-Maddison (SM) and Simmonds Association Index (AI) tests, were performed. The SM test is a tree-based test that determines the minimum number of migration events between the different compartment populations consistent with the structure of the phylogenetic tree [35]. A single or low number of migration events is consistent with compartmentalization, where as multiple migrations would indicate unrestricted flow of quasispecies between the two compartments. The Simmonds AI is another tree-based test that assesses the degree of population structure within the tree by weighting the contribution of each internal node based on its depth in the tree [36]. The AI is the ratio of the association value of test sequences over that of 10 randomly created control sequence sets. There was excellent concordance of the determination of compartmentalization by all three methods (Table 2).

Recombination was assessed as a possible confounding effect on compartmentalization. There was evidence of recombination between plasma and gut isolates only in subject 160. All of the sequences in the highly supported gut compartment contain stretches of sequences derived from at least 2 different plasma isolates (Figure S2), but the recombinant portions did not affect the significant compartmentalization seen for this subject.

### Nef quasispecies in the gut function differently than circulating plasma quasispecies

**Figure 2:**
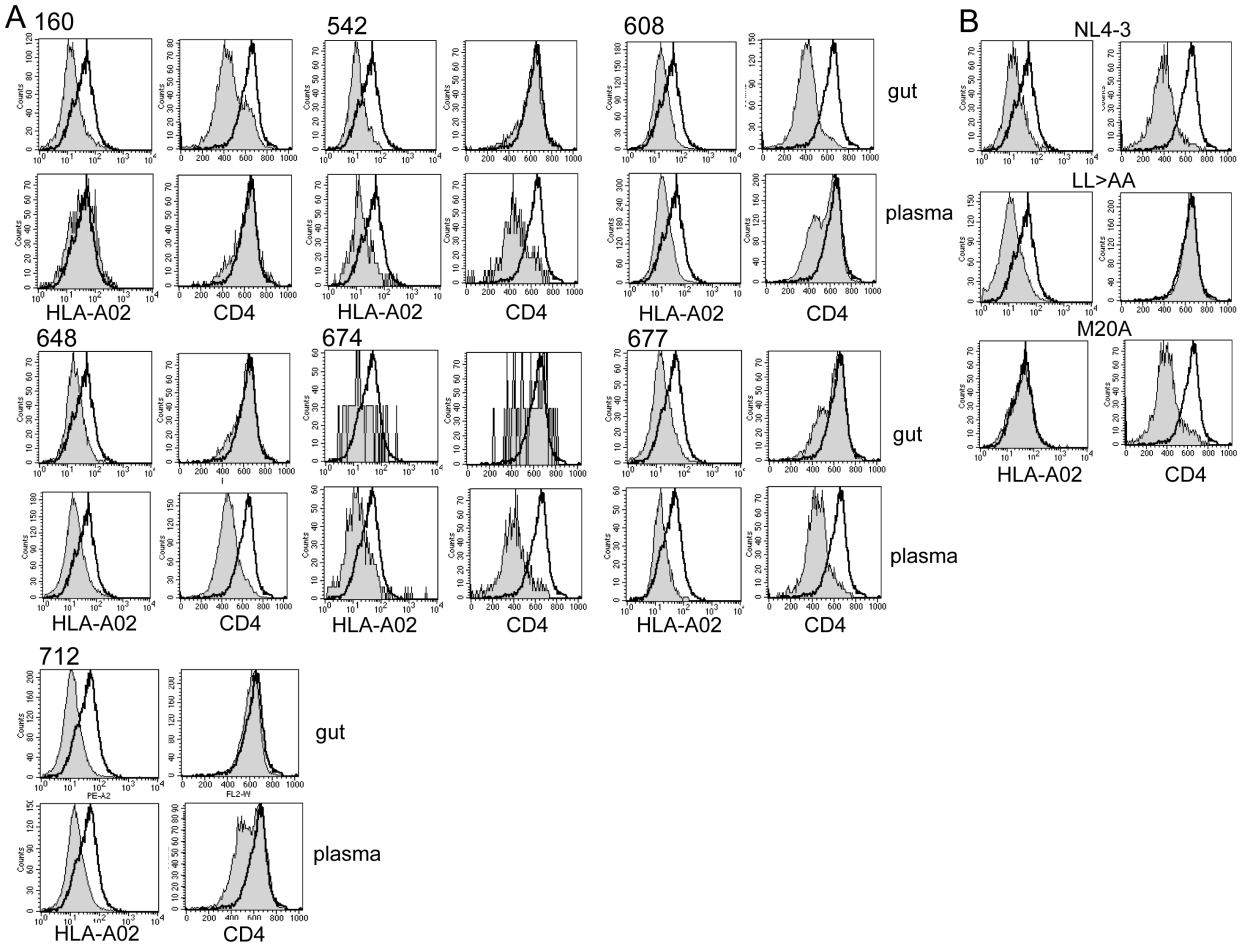
MHC Class I and CD4 downregulation by plasma and gut *nef* quasispecies. VSVg pseudotyped Env^−^ Vpu^−^ recombinant mCD24 reporter viruses were used to infect CEM T1 cells. On day 3 post-infection levels of CD4 and HLA-A*02 were measured on all mCD24 reporter positive cells. Open histograms are Delta Nef control viruses and the filled histograms are the experimental viruses. A.) For each subject 4 histogram plots are shown: HLA-A*02 downregulation by gut and plasma quasispecies on the left panels (upper-gut and lower-plasma) and CD4 downregulation on the right panels (upper-gut and lower-plasma). B.) Downregulation of HLA-A*02 (left) and CD4 (right) by control viruses carrying NL4-3 Nef, Nef LL>AA specifically deficient in CD4 downregulation, and Nef M20A specifically deficient in MHC Class I downregulation.

**Figure 3:**
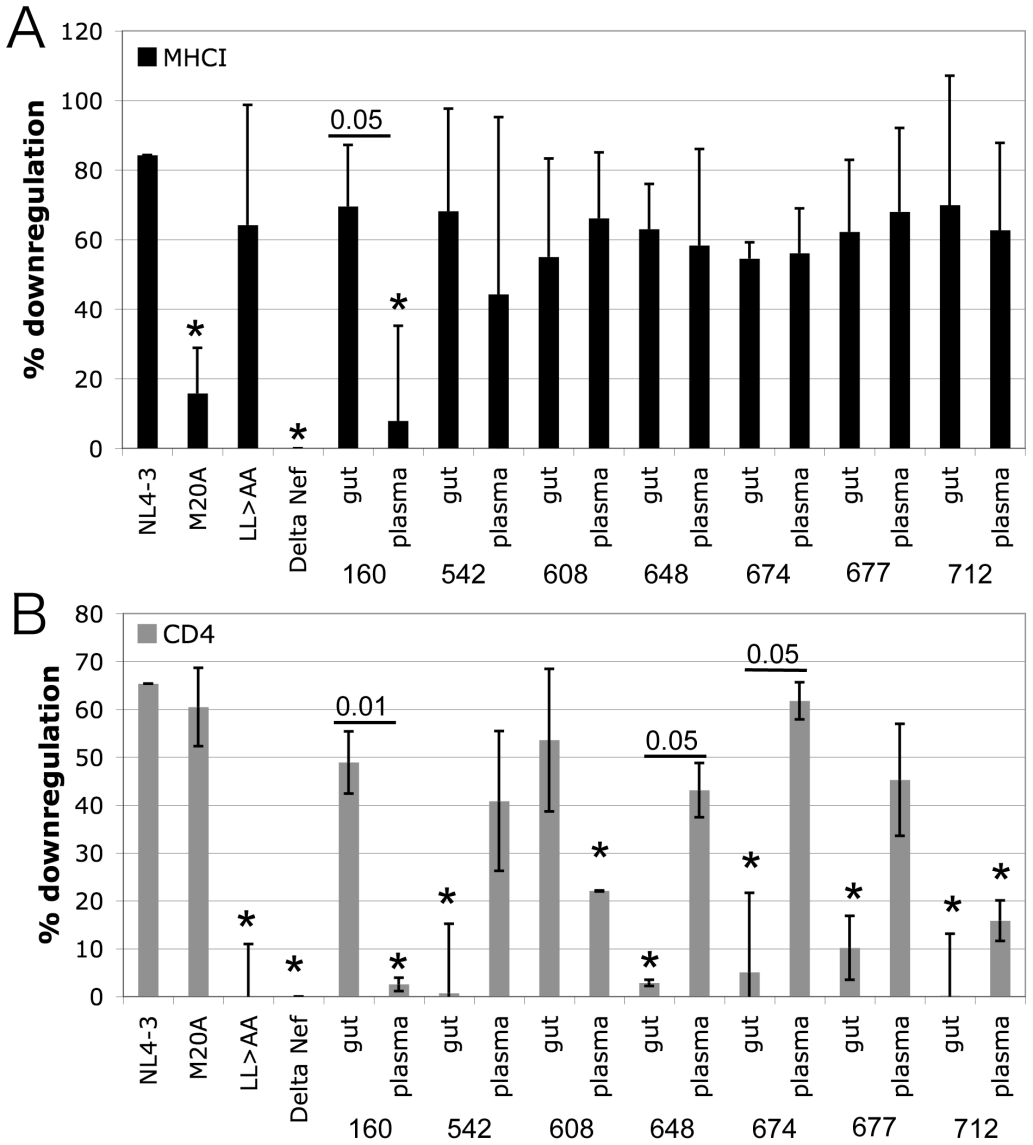
Summary of MHC Class I and CD4 downregulation. The mean and standard deviation of HLA-A*02 (A) and CD4 (B) downregulation by control and subject-derived *nef* alleles from at least three separate infections is shown. Difference from % downregulation by NL4-3 Nef was evaluated with a two-tailed t test, and differences with a p-value <0.05 are marked with an *. Differences between gut and plasma quasispecies from the same subject were also evaluated with a two-tailed t test, and pairs with significant differences are marked with a horizontal bar with the p-value indicated above the bar.

Two key functions of Nef, CD4 and MHC Class I downregulation, were measured by flow cytometry. Total quasispecies mixtures were expressed at physiologic levels under the control of the viral LTR in the context of a proviral clone. The proviral vector is missing portions of *vpu* and *env* that also downregulate CD4 such that exclusively Nef’s contribution to CD4 downregulation is measured in this system [37]. It has been determined previously that circulating plasma quasispecies may have separate populations of functional and non-functional Nef alleles [16], therefore total quasispecies were cloned and tested in bulk (Figure 2). Although most samples had uniform function, several subjects did demonstrate quasispecies populations with a mixture of functional and non-functional alleles (see Figure 2, 608 and 712 plasma and 160 and 677 gut populations). MHC Class I downregulation was relatively well preserved by all quasispecies regardless of location with only subject 160 plasma quasispecies showing significantly diminished function (Figures 2 and 3A). However, there was significant loss of CD4 downregulation by gut quasispecies in 5 of 7 subjects (Figures 2A and 3B). Preservation of the quasispecies diversity during cloning and virus production could not be confirmed for subject 650 and therefore this subject was excluded from subsequent statistical analysis. However, testing individual clones from subject 650 showed that CD4 downregulation was lost by some gut isolates and preserved in all blood isolates tested, while MHC Class I downregulation was relatively preserved in both compartments (Figure S3). Across all subjects there was no concordance between plasma and gut quasispecies function for either CD4 and MHC Class I downregulation (R^2^  =  0.39 p-value 0.13 and R^2^  =  0.26 p-value 0.24, respectively) indicative of compartmentalization of function. Pairwise comparison of function in gut vs. plasma for each subject revealed significant discordance of both MHC Class I and CD4 downregulation for subject 160, and discordance of only CD4 downregulation for subjects 648 and 674 (Figure 3). Subjects 542, 608, 677, and 712 all showed a trend for discordant CD4 downregulation but failed to reach statistical significance (p-value range 0.10–0.17).

Examination of the amino acid sequences of the non-functional quasispecies did not reveal any common substitutions or polymorphisms at sites of known functional significance [38]. Subject 160 plasma sequences did have significant polymorphism changing the EEEE_62−65_ acidic domain to EGGR, which likely affected MHC Class I downregulation, and DD_174−175_ changed to DG, possibly affecting its CD4 downregulation. Otherwise there were only sporadic polymorphisms present in sites not previously associated with Nef function.

### Cytotoxic T lymphocyte responses are not compartmentalized

**Figure 4:**
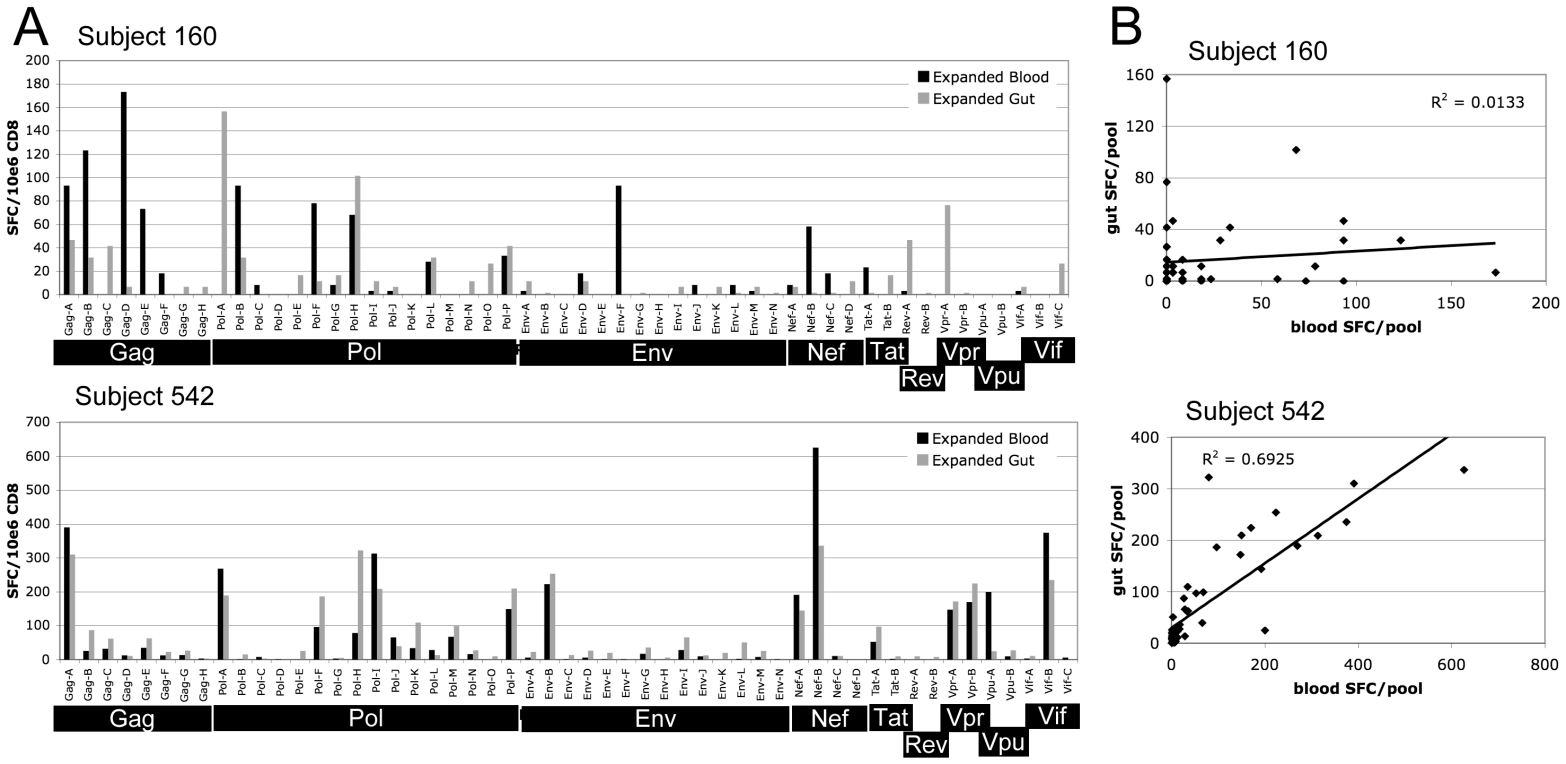
Comparison of CTL responses by CD8+ lymphocytes from PBMC and MMC from gut biopsy specimens. HIV-specific CTL responses were mapped by IFN-gamma ELISpot using expanded CD8+ lymphocytes and pools of Clade B consensus peptides covering the entire HIV proteome. CTL mapping was performed twice with blood and gut biopsy specimens collected on separate visits about 2 weeks apart. A.) ELISpot results showing the mean number of Spot Forming Cells (SFC) per million CD8+ cells for each of the 53 peptide pools. Subject 160 is representative of the 2 subjects with discordant responses in blood and gut. Subject 542 is representative of the remaining 6 subjects with concordant responses. B.) X-Y scatter plot of mean blood vs. gut CTL responses for the same subjects as in A. Regression lines and R^2^ values are shown. The p-value for the positive concordance seen for subject 542 is <0.001. See Table 3 for a summary of all correlation coefficients and p-values for each subject.

CD8+ lymphocytes were expanded from PBMC and from tissue infiltrating mucosal mononuclear cells (MMCs). An IFN-gamma ELISpot assay using 53 pools of Clade B consensus peptides (12–16 peptides/pool) covering the entire HIV-1 proteome was used to screen expanded CD8+ cells for HIV-specific responses. ELISpot assays were performed twice with PBMC and MMC samples collected two weeks apart, and mean Spot Forming Cell (SFC) counts per million CD8+ cells per pool were calculated. Figure 4 shows representative ELISpot results comparing PBMC and MMC HIV-specific responses for two subjects. Concordance between the breadth and magnitude of the CTL response in blood and gut was determined by calculating the Pearson correlation coefficient and performing regression analysis. Table 3 summarizes the correlations coefficients and p-values supporting the overall concordance of CTL responses in blood and gut. Six of eight subjects had strong concordance of blood and gut ELISpot results (correlation coefficients, r, ranging from 0.7069–0.8762 with p-values <0.0001.) as represented by subject 542 in Figure 4. Two subjects did not have concordant responses, represented by subject 160 in Figure 4. In one case (subject 160), discordance was due to a similar overall magnitude of response but with different epitope targeting in the two compartments. In the other case (subject 677), discordance was due to much greater breadth and magnitude of the CTL response in blood relative to gut (Figure S4).

**Table 3:** Concordance of Gut vs. Blood CTL responses.

*Subject number*	*Correlation coefficient^a^*	*p value*
160	0.1189	0.396
542	0.8322	1.142 E-14
608	0.7069	3.283 E-09
648	0.8393	4.163 E-15
650	0.8762	8.485 E-18
674	0.8614	1.268 E-16
677	0.0467	0.739
712	0.7992	7.267 E-13

a Pearson coefficient, R, calculated using regression analysis with

least-squares fit. Total IFN-gamma SFC from blood and gut were compared.

### Selective pressure is similar in gut and plasma compartments

**Figure 5:**
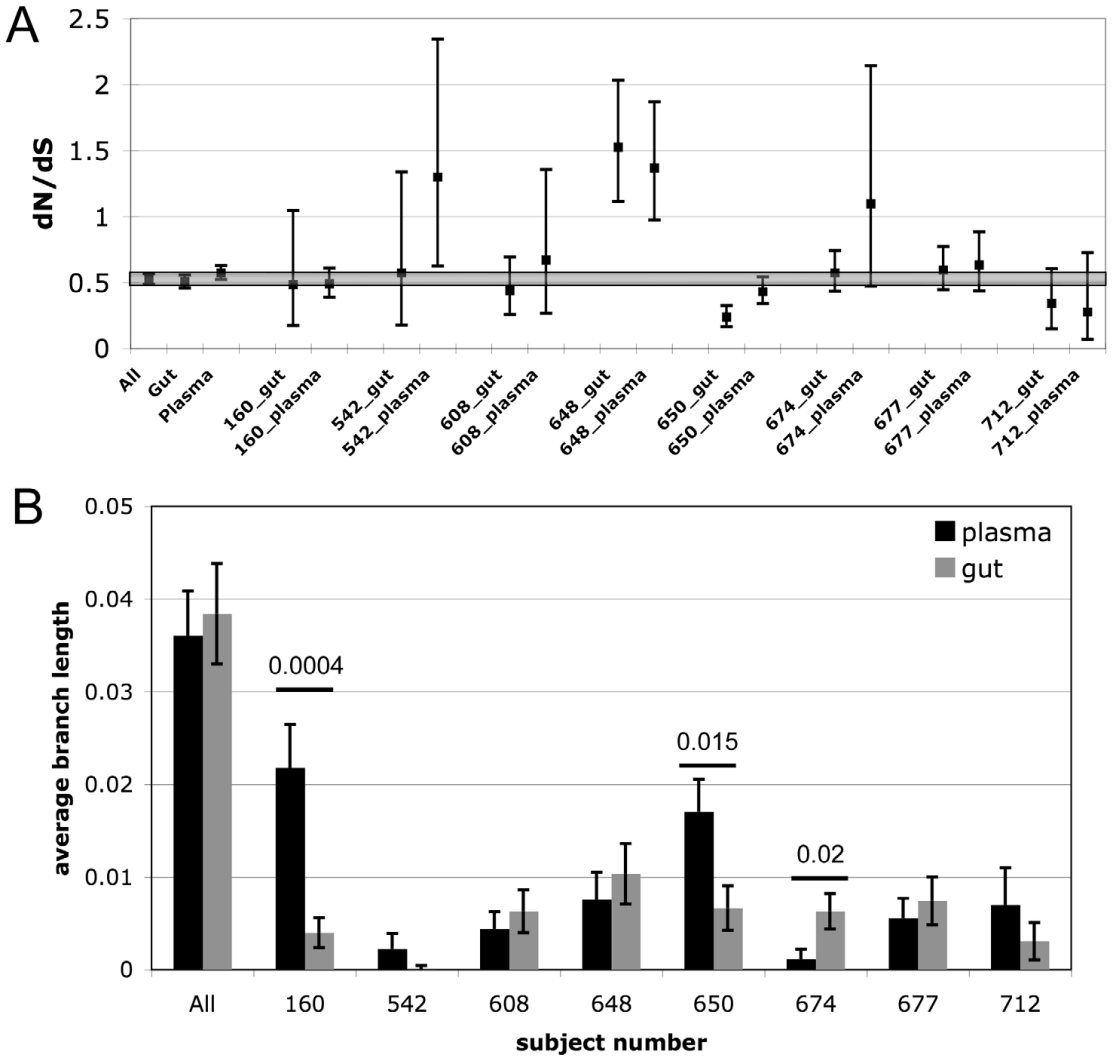
Measures of selective pressure on blood and gut *nef* quasispecies. A.) The global non-synonymous to synonymous substitution rate ratio (dN/dS) with 95% confidence intervals (CI) was calculated for all 234 sequences in the dataset, all gut-derived sequences, all plasma-derived sequences, and for all individual pairs of gut and plasma sequences. The range of the 95% CI for the dN/dS estimate for all sequences is highlighted in grey so that those estimates with a 95% CI entirely outside this range (542 gut, 648 gut and plasma, 650 gut) can be easily identified. B.) The diversity among all sequences from plasma and gut as well as among blood and gut isolates from each subject was calculated with a s.e.m. from 500 bootstrap replicates. The difference in diversity between plasma and gut sequences was evaluated with a two-tailed t test and pairs with significant differences are marked with a bar with the p-value indicated above it.

Multiple measures for evidence of selective pressure were calculated from the sequences obtained from the 2 compartments including diversity, divergence from Clade B consensus, global nonsynonymous to synonymous substitution rate ratio (dN/dS), and site-by-site dN/dS. The dN/dS can be used to measure whether the rate of nonsynonymous substitutions is higher or lower than would be expected from neutral evolution as an indication of the presence of a selective evolutionary force. A dN/dS higher than 1 indicates positive selection, less than one indicates purifying selection. The estimated dN/dS using all sequences from both compartments was 0.525 (95% CI 0.487156-0.565962), consistent with previous reports [38], indicating that Nef is under strong purifying selection. The dN/dS was then estimated for sequences from each compartment for each subject and compared to the dN/dS of all sequences (Figure 5A). Overall there was strong concordance of the dN/dS estimates for gut and plasma sequences (r  =  0.722, R^2^  =  0.522, p  =  0.043) indicating that the selective pressure was uniform between the 2 compartments. Three datasets had significantly higher dN/dS than the whole dataset (542 plasma, 648 gut and plasma), while only one had a dN/dS lower (650 gut). As an additional measure of adaptation the amount of sequence diversity within each compartment was calculated (Figure 5B). Five of eight subjects had no significant differences in diversity between the 2 compartments. Of the three subjects with significant differences in diversity between gut and blood isolates, two had significantly less diversity among the gut-derived sequences, while one had greater diversity among gut sequences. Overall, there was no concordance in the amount of diversity among gut and plasma isolates (p  =  0.9). The difference in concordance of dN/dS and diversity measurements is likely because diversity may largely consist of nonsynonymous substitutions. Only subject 160 had a significant change in divergence from Clade B consensus between the two compartments (Figure 1and Figure S5). Site-by-site calculation of the dN/dS to detect individual amino acid residues under positive or purifying selection did not reveal any codons undergoing compartment-specific selection across the entire dataset. However, the gut isolates from subject 650 did have the highest number of individual sites under strong purifying selection (Figure S6) consistent with the statistically lower diversity and lower dN/dS (Figure 5).

### Amount of selective pressure correlates with the CTL response

**Figure 6:**
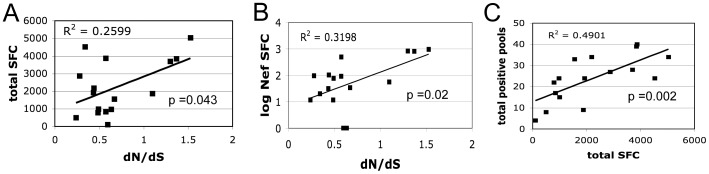
Amount of selective pressure correlates with the CTL response regardless of location. Regression analysis shows a statistically significant correlation between the total mean number of SFC and the estimated global dN/dS from all samples, both blood and gut, for each subject (A). The correlation between CTL and dN/dS from all samples is more significant if only Nef-specific SFCs are considered (B). Finally, there is a significant correlation between the total mean number of SFCs and the number of positive peptide pools (C).

In order to determine the extent to which CTL may be the driving force of *nef* adaptation correlations between measures of selective pressure and CTL responses were calculated. Significant positive correlations were detected between the total number of SFC and the global dN/dS (Figure 6A) and more significantly between total number of Nef-specific SFC and the global dN/dS (Figure 6B) regardless of location. Due to the limited number of cells obtainable from the gut biopsies CTL responses were not mapped to individual 15mers, therefore the number of SFC rather than the number of positive pools is likely a better representation of the magnitude of the CTL response since there may be more than one peptide per pool stimulating a positive IFN-gamma response. However, there was a significant correlation between total SFC and number of positive peptide pools indicating that total SFC reflects the total number of CTL (Figure 6C). Overall, the magnitude of the CTL response correlated with the amount of positive selection regardless of location. Therefore CTL selective pressure is not responsible for the sequence or functional compartmentalization of *nef* quasispecies in the gut in this cohort.

## Discussion

The results presented here indicate that the gut microenvironment promotes selection of a functionally distinct population of viruses, but that CTL did not appear to be the driving force for compartmentalization. Given that CTL appear to be the main force driving evolution of HIV [39], it was somewhat surprising to see no correlation between CTL selective pressure and compartmentalization. However, this perhaps should not have been so unexpected given previous observations that CTL responses are similar between blood and gut compartments [40]. Our data do still support CTL as the main driving force of adaptive evolution overall with the amount of selection correlating with CTL responses. Although the breadth and magnitude of the CTL response is the same in gut and the peripheral blood as enumerated by IFN-γ ELISpot, it may be that the quality or efficacy of the CTL response in the gut may be different if more parameters of CTL function were measured. However, two studies examining multiple functions of CTL including cytokine secretion, degranulation and killing, did not find any significant difference between blood and gut mucosal CTL[41], [42]. Musey et al. reported that CTL clones from blood and mucosa (rectum, cervix, and semen) had similar ability to lyse infected cells in vitro, but there may be additional factors *in vivo* that affect CTL function in different tissue compartments [41]. Ferre et al. compared cytokine secretion and degranulation functions of HIV specific CD8+ cells from blood and rectal mucosa and found no significant difference in function between blood and gut CTLs except in the case of elite controllers, who had more functional gut CTLs [42]. Although the measurement of IFN-γ secretion is not a comprehensive measure of CTL function, in this study of chronically infected individuals it is likely a useful surrogate marker to enumerate HIV-specific CTL responses.

Although we conclude that differential CTL selective pressure does not account for *nef* quasispecies compartmentalization, one subject proved to be an exception. Subject 160 did have evidence of differential CTL targeting together with sequence and functional compartmentalization. This subject had different epitope targeting in gut and plasma and very different sequences in the two compartments with significant differences in diversity and divergence from consensus B. There were several positively selected sites exclusively in plasma isolates associated with significant differences in function; gut isolates were fully functional and plasma isolates, non-functional. Although it was our original hypothesis that CTL selective pressure would lead to compartmentalization of Nef sequence and function, this situation proved to be the exception. This was a small study but still achieved statistically significant correlations indicating that generally differential CTL selective pressure did not drive Nef compartmentalization within gut mucosa.

Studies of compartmentalization must first exclude sampling bias leading to a false positive determination of compartmentalization. Sampling bias due to the very small size of the gut biopsy containing a limited number of infected cells is a significant concern. To control for this possible confounding effect in this study sequences were obtained from multiple different biopsies taken 2 weeks apart. Sequences from the gut from the 2 times points did not form significant independent clusters, rather they were intermingled, suggesting that there was not false positive detection of compartmentalization. Another possible cause of false-positive detection of compartmentalization may be the presence of a population of quasispecies with very low diversity, i.e. – very similar sequences, within the tissue. In their study of genital mucosa isolates Bull et al. reported compartmentalization appeared to be due to low-diversity monotypic populations isolated from mucosa, but this population was not significantly different than peripheral sequences [43]. This was not the case with our subjects where only 2 of 8 had significantly lower diversity in the tissue compartment. In one case, subject 674, there was actually a significant increase in diversity in gut isolates relative to the plasma isolates. Although several subjects had evidence of some monotypic subpopulations, i.e. multiple identical viral sequences, these populations were observed in both gut and blood isolates and in no case did these monotypic populations constitute the entire population from a particular site. Subject 712 did have evidence of low-diversity, monotypic populations in both gut and blood isolates, and although these populations appeared to be separate clusters in the phylogenetic analysis, compartmentalization was rejected by all criteria demonstrating the rigorous standard used for determine compartmentalization in this study. It is also worth noting that with the use of total RNA from the gut biopsies it is not possible to distinguish between virion-derived RNA and intracellular viral RNA. If the intracellular RNA transcripts represented a disproportionate amount of the quasispecies sampled from the gut, then a lower diversity would be expected in gut relative to plasma isolates since the transcripts are all copies from a single genome. However, there is no clear trend for lower gut diversity and several samples have higher diversity in gut relative to plasma. Although it does not appear that the use of both intracellular and cell-free RNA as the source gut-derived sequences has affected the diversity of the quasispecies, it is not possible formally to exclude confounding effects on compartmentalization without separating cell-free viral RNA from the gut biopsies for sequencing.

Previous studies of HIV compartmentalization within the gut have come to different conclusions. Using *env* sequences isolated from proviral DNA from peripheral CD4+ and GALT CD8+ -depleted cells from individuals fully suppressed on antiretroviral therapy Chun et al. reported what they refer to as cross-infection between blood and GALT [31]. They observed little significant change in diversity and multiple migration events between blood and GALT consistent with the conclusion that there is no distinct compartmentalization in aviremic individuals. Similarly, Imamichi et al. also studied *env* sequences from paired blood and gut specimens but from both viremic and aviremic individuals, and they also concluded there was no compartmentalization within the gut [44]. However, van Marle et al. reported significant compartmentalization within the gut when using *nef* sequences isolated from virmeic subjects, similar to what we report here [25]. One likely explanation for the discrepancy of these results is simply that different viral proteins are subject to different structural and functional constraints that shape their evolution within a particular environment. Borderia demonstrated that the degree of tissue compartmentalization is different for *gag* and *pol* coding regions [45]. Given that the amount of positive or purifying selection varies for the different HIV proteins, with Nef generally being under strong purifying selection and Env being under positive selection [46], it may not be so surprising that these two proteins evolve differently within the gut microenvironment. Conclusions about compartmentalization should be viewed with the understanding that viral proteins will each adapt according to their own particular structural and functional constraints. Ultimately, it is important is to consider the functional consequences of local adaptation and not merely the genetic differences between viral isolates in different tissue compartments as small changes in sequence, too small to change the phylogeny, may still be enough to have significant effects on function.

It is important to consider that compartmentalization may be due to differential selective pressure and/or restricted migration between compartments. Although both factors likely play a role in evolution in tissue compartments, the relative balance of these factors may be different. For example, in the case of the CNS restricted migration likely plays a much larger role in compartmentalization compared to the gut. However, in both the gut and the CNS it is likely that Nef compartmentalization is driven by adaptation to selective pressures within the local environment such as availability and permissiveness of CD4+ target cells [22]. The recently published observation that compartmentalization within the female genital track was observed in a cross-sectional study but not longitudinally within individuals supports the idea that compartmentalization may be driven more by ongoing adaptation to local selective pressures and less by restricted trafficking of virus [47]. Over time the population of viruses within a tissue compartment may change, but at any particular moment the population within a compartment such as genital or gut mucosa or CNS may be specifically adapted to local selective pressures, which are distinct from selective forces affecting viruses circulating in the peripheral blood. Our results are not inconsistent with a model of compartmentalization that may be more fluid and transient with the virus quickly adapting as it traffics through a compartment. A longitudinal study would be required to determine whether this is, in fact, the case.

Nef, which has no intrinsic enzymatic activity, has a structure particularly well suited for rapid optimization of its functions with a structured core surrounded by flexible loops that bind numerous cellular factors [48], [49]. Results of recent crystal structures of Nef indicate that it alters its structure depending on its binding partner allowing for the independent optimization of function depending on the local microenvironment [50], [51]. Its flexible structure underlies Nef’s functional plasticity, and it is not difficult to see how small changes in sequence may affect Nef’s function. The trade off between functions by Nef has been well supported with different levels of function at different stages of disease with well-preserved function during transmission/acute infection and near complete loss of function by end-stage disease but variable function during chronic infection [14], [15], [16]. The most obvious difference in Nef function in this study is the loss of CD4 downregulation by the gut quasispecies. This loss of function was not clearly associated with a single common mutation pathway as the differences between functional and non-functional quasispecies were largely sporadic polymorphisms and not in the previously identified functional motifs. This seems to indicate that Nef’s adaptation may take diverse pathways and may require more than one change. In this cross-sectional study it is uncertain whether this is a cause or effect of compartmentalization. It may be that other accessory proteins such as *vpu,* which also downregulates CD4, compensate for this loss of function by Nef freeing up Nef to optimize other functions such as increasing infectivity, T cell activation or viral replication [7]. Work is ongoing to examine these other functions of Nef within the gut mucosa.

Previously we reported a significant correlation between CTL breadth and preservation of Nef-mediated MHC Class I downregulation by plasma quasispecies demonstrating Nef’s adapatation to CTL pressure [16]. In the current study correlations between CTL responses and Nef function did not achieve statistical significance, however, there was a trend (p = 0.07) for a decrease in MHC Class I downregulation correlating with a higher ratio of Nef:non-Nef positive pools. It is likely this did not reach significance both because CTL responses were not mapped to the level of individual peptides hence an accurate number of CTL responses was not known and because the vast majority to isolates in this study had largely preserved the ability to downregulate MHC Class I with very little distribution of the data.

The gut is increasingly gaining attention for it’s potential role in HIV-1 persistence, as it remains a reservoir of viral replication despite fully suppressive antiretroviral therapy [31], [32]. The results presented here indicate that the gut microenvironment promotes selection of a functionally distinct population of viruses, but that CTL, although still the main driver of viral evolution in general, did not appear to be the driving force for compartmentalization. Nef demonstrates genetic and functional compartmentalization within gut mucosa demonstrating its functional plasticity and remarkable adaptability, which may be influenced by factors in addition to CTL responses such as target cell availability. It appears that Nef’s adaptation to the local selective pressures in the gut occurs quickly as there is no evidence of significantly restricted trafficking of CTL or infected cells or virions in and out of the gut mucosa compartment as compared to the central nervous system (CNS). It is not known how compartmentalization within gut mucosa may relate to HIV’s persistence in the gut despite cART. Given our finding of a functionally distinct *nef* quasispecies population in gut and the central roles both Nef and the gut play in pathogenesis it will be important to further investigate Nef’s role in persistent infection in the gut despite cART.

## Materials and Methods

### Ethics statement

All subjects provided written informed consent according to a protocol approved by the UCLA Institutional Review Board. We confirm that this study was specifically approved by the UCLA Institutional Review Board.

### Subject selection and specimen collection

Informed consent was obtained from 8 HIV-1-seropositive individuals not on antiretroviral therapy for at least 12 months through a University of California, Los Angeles, Institutional Review Board-approved study protocol. Peripheral blood and mucosal samples were collected on two consecutive visits 2 weeks apart. Peripheral blood mononuclear cells (PBMCs) were isolated according to a standard Ficoll method. Colonic mucosal mononuclear cells (MMCs) were isolated from multiple tissue biopsies obtained by flexible sigmoidoscopy at approximately 30 cm from the anal verge as previously described [52]. Briefly, tissue-infiltrating lymphocytes were obtained by a combination of collagenase digestion and physical disruption. Typically, this procedure yielded between 2 – 5 million viable CD3+ T lymphocytes per 17 biopsy samples [53]. Nonspecific polyclonal expansion of CD8+ lymphocytes from both PBMCs and MMCs was performed using CD3:CD4-bispecific monoclonal antibodies as previously described [54]. This procedure produced approximately 20×10^6^ cells.

### Viral isolation and nef quasispecies amplification

Viral RNA was isolated from 1 mL of plasma using the UltraSense Viral Isolation kit (Qiagen) according to the manufacturer’s protocol. Total RNA was isolated from at least 2 tissue biopsies from the 2 study visits using a modified TriZol (Invitrogen) extraction protocol as previously described [55]. Note that use of total RNA from the biopsy does not allow for an assessment of whether the RNA is from free virions or intracellular. Total *nef* quasispecies were amplified by a two-step RT-PCR using SuperScriptIII RT kit (Invitrogen) and the high-fidelity polymerase Pfusion (New England Biolabs) with primers and cycle conditions as previously published [38]. Multiple PCRs were performed on each sample then pooled together prior to cloning.

### Cloning and reporter virus production

Bulk *nef* amplification products were cloned into plasmid AA1305#18, an NL4-3-based proviral vector into which the reporter gene murine CD24 has been inserted into *vpr* and portions of *vpu* and *env* also known to contribute to CD4 downregulation have been deleted, as previously described [37]. 293T cells were co-transfected with the AA1305 proviral contructs and a Vesicular Stomatitis Virus glycoprotein (VSVg)-encoding construct to produce pseudotyped recombinant reporter viruses. Cloning efficiencies of 85% or higher were confirmed by plating a portion of the cloning mix and selecting 10–12 colonies to check for the proper restriction digest pattern. Preservation of the diversity of the quasispecies mixture after cloning and virus production was assessed by sequencing virus stocks and quantifying polymorphic positions by examination of electropherograms.

### Sequencing and sequence analysis

Multiple individual clones for each sample were isolated and sequenced. Sequences were aligned with NL4-3 *nef* and the Los Alamos National Laboratory (LANL) HIV-1 database Clade B Consensus *nef* then manually edited by toggling the amino acid translation using the program BioEdit. All sequences were examined for G to A hypermutation using Hypermut 2.0 from the LANL HIV-1 database tools. Sequences were examined for evidence of recombination using SimPlot and Bootscan [56]. Sequences with non-intact reading frames due to frame shift or non-sense mutations were excluded prior to the analysis for adaptive evolution. Phylogenetic trees were constructed with neighbor-joining (NJ) and maximum likelihood (ML) algorithms using PHYLIP 3.64 [57]. The neighbor-joining tree was statistically evaluated with 1000 bootstrap replicates. In addition to determining independent clustering of gut and plasma isolates, the Slatkin Maddison (SM) number of migrations [35] and Simmonds Association Index (AI) [36] tests for compartmentalization as implemented in HyPhy [58] were performed. The probability value for the SM test was set at >90%. Sequence diversity within the quasispecies swarm and overall divergence from Clade B consensus sequence were determined using the program SENDBS with the Hasegawa model + gamma and standard errors estimated from 500 bootstrap replicates. All of the following analyses were performed using HyPhy. The program MODELTEST was used to determine the best fitting model for the data was HKY85. The global dN/dS ratio along with its 95% confidence intervals were estimated after building and optimizing the maximum likelihood function for both gut and plasma sequence data sets. Individual amino acid positions with evidence of adaptive evolution were identified by three separate methods, ancestor counting (SLAC), relative-effects likelihood (REL), and fixed-effects likelihood (FEL). A site was considered to be adapting under differential selective pressure if that site was identified by at least 2 of 3 methods with a significance level of at least 95% and was only identified in one of the two compartments (gut or plasma). Additionally, only those sites with a dN/dS significantly > and < 1 were considered positive. Sequence accession numbers KF313570-KF313803.

### ELISpot mapping

Gamma Interferon (IFN-γ) enzyme-linked immunospot (ELISpot) assay was performed with expanded CD8+ PBMC and expanded CD8+ MMC as previously described [53]. Briefly, CD8+ T lymphocytes were plated at 2×10^5^ to 3×10^5^ cells/well and exposed to a library of HIV-1 peptides (consecutive 15-mers overlapping by 11 amino acids) spanning all HIV-1 proteins, obtained from the NIH AIDS Research and Reference Reagent Repository (all clade B consensus sequences with the exception of Env). Peptides were screened in 53 pools of 12 to 16 peptides each and added to the wells at a final concentration of 5 µg/ml for each individual peptide. Individual IFN-γ -secreting cells (spot-forming cells [SFC]) were counted using an automated ELISpot counting system (Cellular Technologies Limited, Cleveland, Ohio). Assays were performed twice with samples collected 2 weeks apart. Assays with a negative control background mean of less than 100 SFC/10^6^ cells were considered valid. A positive response was defined as a mean being higher than four times the mean of the negative controls or at least 10 SFC/10^6^ cells, whichever was higher.

### Measuring CD4 and HLA downregulation

As previously described [37], CEM T1 cells were infected with VSVg pseudotyped reporter viruses carrying gut- or plasma-derived *nef* quasispecies or the following control *nef* alleles; “wild type” NL4-3, M20A Nef specifically deficient in MHC Class I downregulation [59], LL>AA Nef specifically deficient in CD4 downregulation [60], and Delta Nef. Note that we have previously determined that measuring function of the bulk quasispecies yields results comparable to measuring multiple individual clones[37]. On day 3 post-infection 2×10^5^ cells were stained with anti-murine CD24/HSA-FITC (BD Pharmingen), anti-human CD4-APC (BD Pharmingen) and anti-human HLA A*02-PE (ProImmune). At least 5×10^4^ live cells were counted using a FACScan flow cytometer, and data were analyzed using CellQuest software (Becton Dickinson). Maximum levels of HLA A*02 and CD4 were determined using the Delta Nef mutant. All infections and flow cytometry were performed in triplicate. Significant differences from NL4-3 Nef or between pairs of gut and plasma samples were determined using a two-tailed t test with unequal variance.

### Statistical Analysis

Analysis for concordance of CTL responses in blood vs. gut and for Nef function by blood vs. gut quasispecies was performed by calculating the Pearson correlation coefficient (R) and regression analysis with least squares fit method. Analysis for correlations between the following pairs of observations was performed by using regression analysis: 1.) Nef function vs. CTL magnitude, 2.) Nef function vs. CTL breadth, 3.) Nef function vs. dN/dS, 4.) Nef function vs. total number of positively- or negatively-selected sites, 5.) Nef function vs. Diversity, 6.) CTL magnitude vs. dN/dS, and 7.) CTL breadth vs. dN/dS.

## Supporting Information

Figure S1 **Maximum likelihood phylogenetic tree of plasma and gut-derived** ***nef*** **quasispecies.** 234 full-length *nef* sequences were aligned with consensus B and NL4-3. A maximum likelihood tree was constructed and rooted with consensus B Nef. All clusters with significant bootstrap support in the NJ tree were also significant in the ML tree. Sequences from gut are indicated with black; plasma, grey.
(TIF)

Figure S2 **Bootscanning plot shows recombination between gut and plasma sequences in subject 160.** The consensus of all gut sequences was compared with individual plasma sequences. Bootscanning with a window size of 80nt and a step size of 10nt shows 2 regions with significant bootstrap support (>70%) for recombinant segments within the gut consensus. The first segment from nt 1-60 is a recombinant with plasma clone7, and the second segment from nt 475- 575, with plasma clone 4.
(TIFF)

Figure S3 **Downregulation by individual clones from subject 650 indicates two different functional populations in the gut.** Unfortunately preservation of quasispecies diversity during cloning and virus production could not be confirmed for subject 650. Therefore individual clones were tested for the ability to downregulate MHC Class I (black bars) and CD4 (grey bars). Levels of of HLA-A*02 and CD4 were measured by flow cytometry 3 days after infection of CEM T1 cells with viruses encoding individual plasma- or gut-derived Nef (plasma 1 and 2, gut 1 and 2) or control viruses carrying NL4-3 Nef, Nef LL>AA specifically deficient in CD4 downregulation, and Nef M20A specifically deficient in MHC Class I downregulation. Percent downregulation was determined using Delta Nef and isotype controls. Shown are the average results of 3 separate infections.
(TIFF)

Figure S4 **Subject 677 has discordant CTL responses in PBMC vs. MMC.** HIV-specific CTL responses were mapped by IFN-gamma ELISpot using expanded CD8+ lymphocytes and pools of Clade B consensus peptides covering the entire HIV proteome. CTL mapping was performed twice with blood and gut biopsy specimens collected on separate visits about 2 weeks apart. ELISpot results showing the mean number of Spot Forming Cells (SFC) per million CD8+ cells for each of the 53 peptide pools. Subject 677 had significantly more and higher magnitude responses from PBMC compared to MMC.
(TIF)

Figure S5 **Only subject 160 had a significant difference in divergence from Consensus B between plasma and gut sequences.** The divergence of plasma and gut sequences from Consensus B was calculated along with a s.e.m. from 500 bootstrap replicates. The difference in divergence between plasma and gut sequences was evaluated with a two-tailed t test and pairs with significant differences are marked with a bar with the p-value indicated above it.
(TIF)

Figure S6 **Number of individually selected sites in plasma and gut sequences.** Individual amino acid positions with evidence of adaptive evolution were identified by three separate methods, and a site was considered to be adapting under differential selective pressure if that site was identified by at least 2 of 3 methods with a significance level of at least 95% and was only identified in one of the two compartments (gut or plasma). Additionally, only those sites with a dN/dS significantly > and < 1 were considered positive. Subject 650 gut sequences had the highest number of site under purifying selection, while several samples (160gut, 542 plasma, 608 gut and plasma, 712 gut and plasma) had no sites undergoing significant adaptive evoltion.
(TIF)

## References

[1] Jamieson BD, Aldrovandi GM, Planelles V, Jowett JB, Gao L, et al (1994) Requirement of human immunodeficiency virus type 1 nef for in vivo replication and pathogenicity. J Virol 68: 3478–3485. 8189487 10.1128/jvi.68.6.3478-3485.1994 PMC236850

[2] Swigut T, Alexander L, Morgan J, Lifson J, Mansfield KG, et al (2004) Impact of Nef-mediated downregulation of major histocompatibility complex class I on immune response to simian immunodeficiency virus. J Virol 78: 13335–13344. 15542684 10.1128/JVI.78.23.13335-13344.2004 PMC525019

[3] Daniel MD, Kirchhoff F, Czajak SC, Sehgal PK, Desrosiers RC (1992) Protective effects of a live attenuated SIV vaccine with a deletion in the nef gene. Science 258: 1938–1941. 1470917 10.1126/science.1470917

[4] Dyer WB, Geczy AF, Kent SJ, McIntyre LB, Blasdall SA, et al (1997) Lymphoproliferative immune function in the Sydney Blood Bank Cohort, infected with natural nef/long terminal repeat mutants, and in other long-term survivors of transfusion-acquired HIV-1 infection. Aids 11: 1565–1574. 9365760 10.1097/00002030-199713000-00004

[5] Deacon NJ, Tsykin A, Solomon A, Smith K, Ludford-Menting M, et al (1995) Genomic structure of an attenuated quasi species of HIV-1 from a blood transfusion donor and recipients. Science 270: 988–991. 7481804 10.1126/science.270.5238.988

[6] Oelrichs R, Tsykin A, Rhodes D, Solomon A, Ellett A, et al (1998) Genomic sequence of HIV type 1 from four members of the Sydney Blood Bank Cohort of long-term nonprogressors. AIDS Res Hum Retroviruses 14: 811–814. 9643382 10.1089/aid.1998.14.811

[7] Kirchhoff F (2010) Immune evasion and counteraction of restriction factors by HIV-1 and other primate lentiviruses. Cell Host Microbe 8: 55–67. 20638642 10.1016/j.chom.2010.06.004

[8] Geffin R, Wolf D, Muller R, Hill MD, Stellwag E, et al (2000) Functional and structural defects in HIV type 1 nef genes derived from pediatric long-term survivors. AIDS Res Hum Retroviruses 16: 1855–1868. 11118071 10.1089/08892220050195810

[9] Collins KL, Chen BK, Kalams SA, Walker BD, Baltimore D (1998) HIV-1 Nef protein protects infected primary cells against killing by cytotoxic T lymphocytes. Nature 391: 397–401. 9450757 10.1038/34929

[10] Schwartz O, Marechal V, Le Gall S, Lemonnier F, Heard JM (1996) Endocytosis of major histocompatibility complex class I molecules is induced by the HIV-1 Nef protein. Nat Med 2: 338–342. 8612235 10.1038/nm0396-338

[11] Garcia JV, Miller AD (1991) Serine phosphorylation-independent downregulation of cell-surface CD4 by nef. Nature 350: 508–511. 2014052 10.1038/350508a0

[12] Lama J, Mangasarian A, Trono D (1999) Cell-surface expression of CD4 reduces HIV-1 infectivity by blocking Env incorporation in a Nef- and Vpu-inhibitable manner. Curr Biol 9: 622–631. 10375528 10.1016/s0960-9822(99)80284-x

[13] Yang OO, Nguyen PT, Kalams SA, Dorfman T, Gottlinger HG, et al (2002) Nef-mediated resistance of human immunodeficiency virus type 1 to antiviral cytotoxic T lymphocytes. J Virol 76: 1626–1631. 11799157 10.1128/JVI.76.4.1626-1631.2002 PMC135916

[14] Noviello CM, Pond SL, Lewis MJ, Richman DD, Pillai SK, et al (2007) Maintenance of Nef-mediated modulation of major histocompatibility complex class I and CD4 after sexual transmission of human immunodeficiency virus type 1. J Virol 81: 4776–4786. 17329339 10.1128/JVI.01793-06 PMC1900175

[15] Carl S, Greenough TC, Krumbiegel M, Greenberg M, Skowronski J, et al (2001) Modulation of different human immunodeficiency virus type 1 Nef functions during progression to AIDS. J Virol 75: 3657–3665. 11264355 10.1128/JVI.75.8.3657-3665.2001 PMC114857

[16] Lewis MJ, Balamurugan A, Ohno A, Kilpatrick S, Ng HL, et al (2008) Functional adaptation of Nef to the immune milieu of HIV-1 infection in vivo. J Immunol 180: 4075–4081. 18322217 10.4049/jimmunol.180.6.4075

[17] Diem K, Nickle DC, Motoshige A, Fox A, Ross S, et al (2008) Male genital tract compartmentalization of human immunodeficiency virus type 1 (HIV). AIDS Res Hum Retroviruses 24: 561–571. 18426336 10.1089/aid.2007.0115

[18] Kemal KS, Foley B, Burger H, Anastos K, Minkoff H, et al (2003) HIV-1 in genital tract and plasma of women: compartmentalization of viral sequences, coreceptor usage, and glycosylation. Proc Natl Acad Sci U S A 100: 12972–12977. 14557540 10.1073/pnas.2134064100 PMC240729

[19] Bull ME, Learn GH, McElhone S, Hitti J, Lockhart D, et al (2009) Monotypic human immunodeficiency virus type 1 genotypes across the uterine cervix and in blood suggest proliferation of cells with provirus. J Virol 83: 6020–6028. 19339344 10.1128/JVI.02664-08 PMC2687376

[20] Salemi M, Lamers SL, Yu S, de Oliveira T, Fitch WM, et al (2005) Phylodynamic analysis of human immunodeficiency virus type 1 in distinct brain compartments provides a model for the neuropathogenesis of AIDS. J Virol 79: 11343–11352. 16103186 10.1128/JVI.79.17.11343-11352.2005 PMC1193641

[21] Ritola K, Robertson K, Fiscus SA, Hall C, Swanstrom R (2005) Increased human immunodeficiency virus type 1 (HIV-1) env compartmentalization in the presence of HIV-1-associated dementia. J Virol 79: 10830–10834. 16051875 10.1128/JVI.79.16.10830-10834.2005 PMC1182623

[22] Olivieri KC, Agopian KA, Mukerji J, Gabuzda D (2010) Evidence for adaptive evolution at the divergence between lymphoid and brain HIV-1 nef genes. AIDS Res Hum Retroviruses 26: 495–500. 20377428 10.1089/aid.2009.0257 PMC2933169

[23] de Oliveira T, Lamers SL, Salemi M, McGrath MS (2008) Compartmentalizatin and Evolutionary Dynamics of HIV-1 Nef Sequences in the Brain of 5 Individuals. 15th Conference on Retroviruses and Opportunistic Infections Boston.

[24] Gray LR, Gabuzda D, Cowley D, Ellett A, Chiavaroli L, et al (2011) CD4 and MHC class 1 down-modulation activities of nef alleles from brain- and lymphoid tissue-derived primary HIV-1 isolates. J Neurovirol 17: 82–91. 21165790 10.1007/s13365-010-0001-6 PMC4395859

[25] van Marle G, Gill MJ, Kolodka D, McManus L, Grant T, et al (2007) Compartmentalization of the gut viral reservoir in HIV-1 infected patients. Retrovirology 4: 87. 18053211 10.1186/1742-4690-4-87 PMC2217557

[26] Brenchley JM, Schacker TW, Ruff LE, Price DA, Taylor JH, et al (2004) CD4+ T cell depletion during all stages of HIV disease occurs predominantly in the gastrointestinal tract. J Exp Med 200: 749–759. 15365096 10.1084/jem.20040874 PMC2211962

[27] Schneider T, Jahn HU, Schmidt W, Riecken EO, Zeitz M, et al (1995) Loss of CD4 T lymphocytes in patients infected with human immunodeficiency virus type 1 is more pronounced in the duodenal mucosa than in the peripheral blood. Berlin Diarrhea/Wasting Syndrome Study Group. Gut 37: 524–529. 7489940 10.1136/gut.37.4.524 PMC1382905

[28] Shacklett BL (2010) Immune responses to HIV and SIV in mucosal tissues: 'location, location, location'. Curr Opin HIV AIDS 5: 128–134. 20543589 10.1097/COH.0b013e328335c178 PMC2886278

[29] Picker LJ, Hansen SG, Lifson JD (2012) New paradigms for HIV/AIDS vaccine development. Annu Rev Med 63: 95–111. 21942424 10.1146/annurev-med-042010-085643 PMC3368276

[30] Lehner T, Anton PA (2002) Mucosal immunity and vaccination against HIV. Aids 16 Suppl 4S125–132. 12699009 10.1097/00002030-200216004-00017

[31] Chun TW, Nickle DC, Justement JS, Meyers JH, Roby G, et al (2008) Persistence of HIV in gut-associated lymphoid tissue despite long-term antiretroviral therapy. J Infect Dis 197: 714–720. 18260759 10.1086/527324

[32] Poles MA, Boscardin WJ, Elliott J, Taing P, Fuerst MM, et al (2006) Lack of decay of HIV-1 in gut-associated lymphoid tissue reservoirs in maximally suppressed individuals. J Acquir Immune Defic Syndr 43: 65–68. 16936559 10.1097/01.qai.0000230524.71717.14

[33] Brenchley JM, Price DA, Schacker TW, Asher TE, Silvestri G, et al (2006) Microbial translocation is a cause of systemic immune activation in chronic HIV infection. Nat Med 12: 1365–1371. 17115046 10.1038/nm1511

[34] Zarate S, Pond SL, Shapshak P, Frost SD (2007) Comparative study of methods for detecting sequence compartmentalization in human immunodeficiency virus type 1. J Virol 81: 6643–6651. 17428864 10.1128/JVI.02268-06 PMC1900087

[35] Slatkin M, Maddison WP (1989) A cladistic measure of gene flow inferred from the phylogenies of alleles. Genetics 123: 603–613. 2599370 10.1093/genetics/123.3.603 PMC1203833

[36] Wang TH, Donaldson YK, Brettle RP, Bell JE, Simmonds P (2001) Identification of shared populations of human immunodeficiency virus type 1 infecting microglia and tissue macrophages outside the central nervous system. J Virol 75: 11686–11699. 11689650 10.1128/JVI.75.23.11686-11699.2001 PMC114755

[37] Ali A, Realegeno S, Yang OO, Lewis MJ (2009) Simultaneous assessment of CD4 and MHC-I downregulation by Nef primary isolates in the context of infection. J Virol Methods 161: 297–304. 19643141 10.1016/j.jviromet.2009.07.006 PMC2758048

[38] Lewis MJ, Lee P, Ng HL, Yang OO (2012) Immune selection in vitro reveals human immunodeficiency virus type 1 Nef sequence motifs important for its immune evasion function in vivo. J Virol 86: 7126–7135. 22553319 10.1128/JVI.00878-12 PMC3416312

[39] Allen TM, Altfeld M, Geer SC, Kalife ET, Moore C, et al (2005) Selective escape from CD8+ T-cell responses represents a major driving force of human immunodeficiency virus type 1 (HIV-1) sequence diversity and reveals constraints on HIV-1 evolution. J Virol 79: 13239–13249. 16227247 10.1128/JVI.79.21.13239-13249.2005 PMC1262562

[40] Ibarrondo FJ, Anton PA, Fuerst M, Ng HL, Wong JT, et al (2005) Parallel human immunodeficiency virus type 1-specific CD8+ T-lymphocyte responses in blood and mucosa during chronic infection. J Virol 79: 4289–4297. 15767429 10.1128/JVI.79.7.4289-4297.2005 PMC1061549

[41] Musey L, Ding Y, Cao J, Lee J, Galloway C, et al (2003) Ontogeny and specificities of mucosal and blood human immunodeficiency virus type 1-specific CD8(+) cytotoxic T lymphocytes. J Virol 77: 291–300. 12477834 10.1128/JVI.77.1.291-300.2003 PMC140595

[42] Ferre AL, Hunt PW, Critchfield JW, Young DH, Morris MM, et al (2009) Mucosal immune responses to HIV-1 in elite controllers: a potential correlate of immune control. Blood 113: 3978–3989. 19109229 10.1182/blood-2008-10-182709 PMC2673124

[43] Bull M, Learn G, Genowati I, McKernan J, Hitti J, et al (2009) Compartmentalization of HIV-1 within the female genital tract is due to monotypic and low-diversity variants not distinct viral populations. PLoS One 4: e7122. 19771165 10.1371/journal.pone.0007122 PMC2741601

[44] Imamichi H, Degray G, Dewar RL, Mannon P, Yao M, et al (2011) Lack of compartmentalization of HIV-1 quasispecies between the gut and peripheral blood compartments. J Infect Dis 204: 309–314. 21673043 10.1093/infdis/jir259 PMC3114472

[45] Borderia AV, Codoner FM, Sanjuan R (2007) Selection promotes organ compartmentalization in HIV-1: evidence from gag and pol genes. Evolution 61: 272–279. 17348938 10.1111/j.1558-5646.2007.00025.x

[46] de Oliveira T, Salemi M, Gordon M, Vandamme AM, van Rensburg EJ, et al (2004) Mapping sites of positive selection and amino acid diversification in the HIV genome: an alternative approach to vaccine design? Genetics 167: 1047–1058. 15280222 10.1534/genetics.103.018135 PMC1470929

[47] Bull ME, Heath LM, McKernan-Mullin JL, Kraft KM, Acevedo L, et al (2013) Human immunodeficiency viruses appear compartmentalized to the female genital tract in cross-sectional analyses but genital lineages do not persist over time. J Infect Dis 207: 1206–1215. 23315326 10.1093/infdis/jit016 PMC3603533

[48] Franken P, Arold S, Padilla A, Bodeus M, Hoh F, et al (1997) HIV-1 Nef protein: purification, crystallizations, and preliminary X-ray diffraction studies. Protein Sci 6: 2681–2683. 9416624 10.1002/pro.5560061227 PMC2143629

[49] Lee CH, Saksela K, Mirza UA, Chait BT, Kuriyan J (1996) Crystal structure of the conserved core of HIV-1 Nef complexed with a Src family SH3 domain. Cell 85: 931–942. 8681387 10.1016/s0092-8674(00)81276-3

[50] Jia X, Singh R, Homann S, Yang H, Guatelli J, et al (2012) Structural basis of evasion of cellular adaptive immunity by HIV-1 Nef. Nat Struct Mol Biol 19: 701–706. 22705789 10.1038/nsmb.2328 PMC3407041

[51] Alvarado JJ, Tarafdar S, Smithgall T, Yeh J (2013) Crystal Structure of HIV-1 Nef in Complex with the Hck SH3-SH2 Region Reveals New Intermolecular Interactions with Functional Implications. 20th Conference on Retroviruses and Opportunistic Infections. Atlanta, Georgia, U.S.A.

[52] Anton PA, Elliott J, Poles MA, McGowan IM, Matud J, et al (2000) Enhanced levels of functional HIV-1 co-receptors on human mucosal T cells demonstrated using intestinal biopsy tissue. Aids 14: 1761–1765. 10985313 10.1097/00002030-200008180-00011

[53] Shacklett BL, Yang O, Hausner MA, Elliott J, Hultin L, et al (2003) Optimization of methods to assess human mucosal T-cell responses to HIV infection. J Immunol Methods 279: 17–31. 12969544 10.1016/s0022-1759(03)00255-2

[54] Wong JT, Colvin RB (1991) Selective reduction and proliferation of the CD4+ and CD8+ T cell subsets with bispecific monoclonal antibodies: evidence for inter-T cell-mediated cytolysis. Clin Immunol Immunopathol 58: 236–250. 1824687 10.1016/0090-1229(91)90139-2

[55] Anton PA, Poles MA, Elliott J, Mao SH, McGowan I, et al (2001) Sensitive and reproducible quantitation of mucosal HIV-1 RNA and DNA viral burden in patients with detectable and undetectable plasma viral HIV-1 RNA using endoscopic biopsies. J Virol Methods 95: 65–79. 11377714 10.1016/s0166-0934(01)00295-6

[56] Lole KS, Bollinger RC, Paranjape RS, Gadkari D, Kulkarni SS, et al (1999) Full-length human immunodeficiency virus type 1 genomes from subtype C-infected seroconverters in India, with evidence of intersubtype recombination. J Virol 73: 152–160. 9847317 10.1128/jvi.73.1.152-160.1999 PMC103818

[57] Felsenstein J (1989) PHYLIP-Phylogeny Inference Package (Version 3.2). Cladistics 5.

[58] Pond SL, Frost SD, Muse SV (2005) HyPhy: hypothesis testing using phylogenies. Bioinformatics 21: 676–679. 15509596 10.1093/bioinformatics/bti079

[59] Akari H, Arold S, Fukumori T, Okazaki T, Strebel K, et al (2000) Nef-induced major histocompatibility complex class I down-regulation is functionally dissociated from its virion incorporation, enhancement of viral infectivity, and CD4 down-regulation. J Virol 74: 2907–2912. 10684310 10.1128/jvi.74.6.2907-2912.2000 PMC111784

[60] Craig HM, Pandori MW, Guatelli JC (1998) Interaction of HIV-1 Nef with the cellular dileucine-based sorting pathway is required for CD4 down-regulation and optimal viral infectivity. Proc Natl Acad Sci U S A 95: 11229–11234. 9736718 10.1073/pnas.95.19.11229 PMC21624

