# Supplementary figures and images for: HIV-1 Nef Sequence and Functional Compartmentalization in the Gut Is Not Due to Differential Cytotoxic T Lymphocyte Selective Pressure
Source: PLoS One. 2013 Sep 13;8(9):e75620. doi: 10.1371/journal.pone.0075620 (PMC3772905; doi:10.1371/journal.pone.0075620)

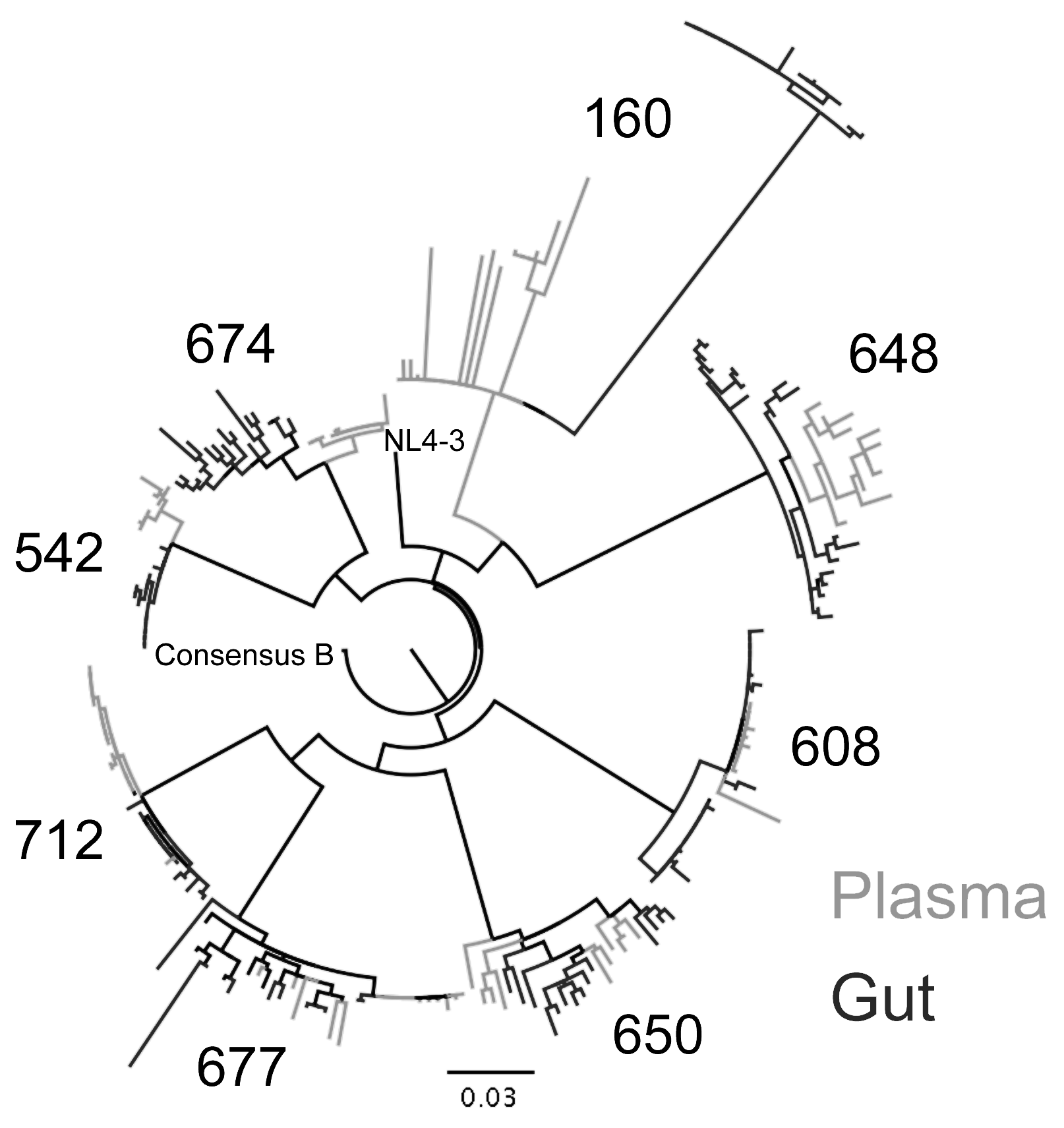

Supplement: Figure S1 — Maximum likelihood phylogenetic tree of plasma and gut-derived nef quasispecies. 234 full-length nef sequences were aligned with consensus B and NL4-3. A maximum likelihood tree was constructed and rooted with consensus B Nef. All clusters with significant bootstrap support in the NJ tree were also significant in the ML tree. Sequences from gut are indicated with black; plasma, grey. (TIF) [file pone.0075620.s001.tif]

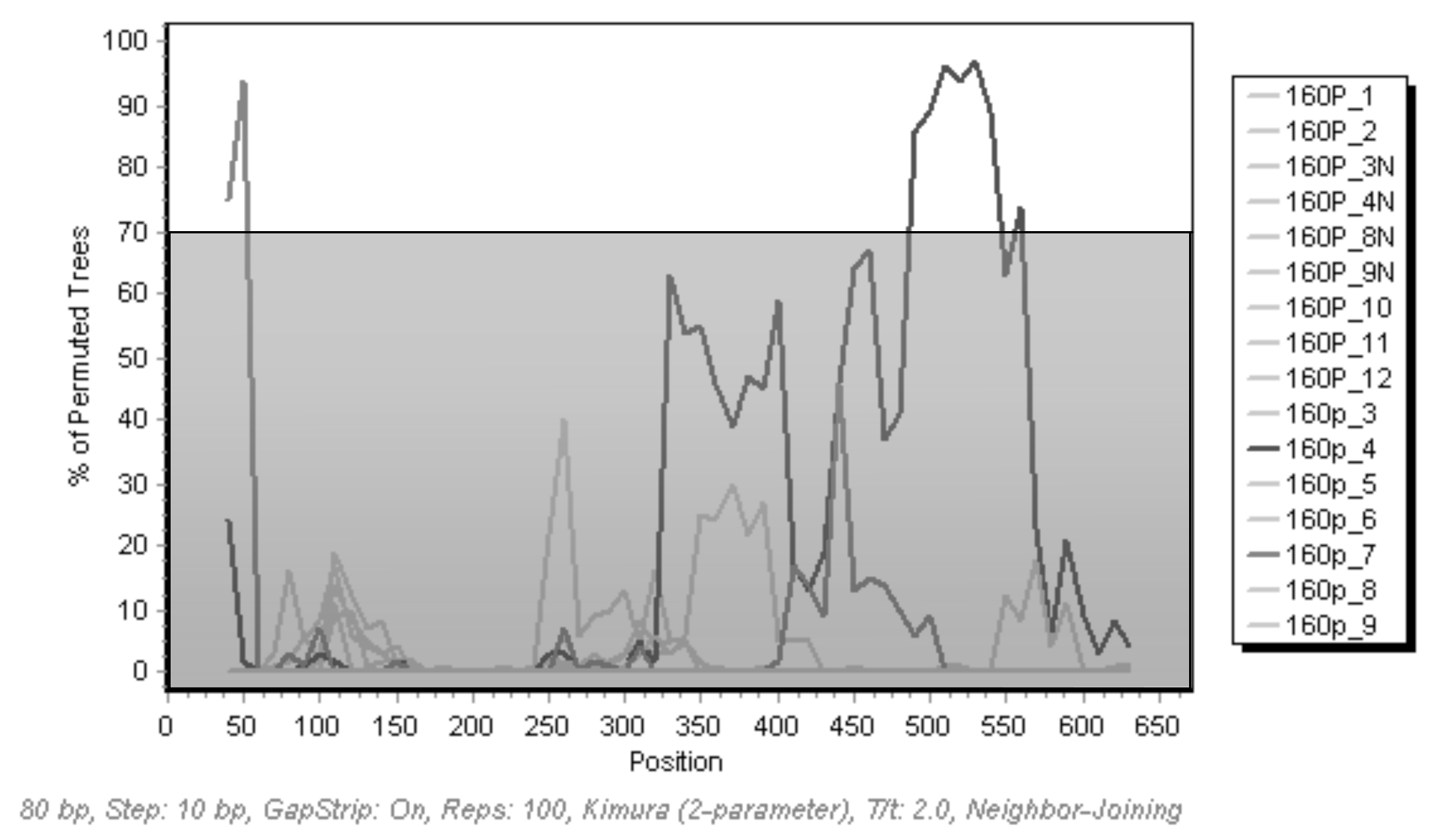

Supplement: Figure S2 — Bootscanning plot shows recombination between gut and plasma sequences in subject 160. The consensus of all gut sequences was compared with individual plasma sequences. Bootscanning with a window size of 80nt and a step size of 10nt shows 2 regions with significant bootstrap support (>70%) for recombinant segments within the gut consensus. The first segment from nt 1-60 is a recombinant with plasma clone7, and the second segment from nt 475- 575, with plasma clone 4. (TIFF) [file pone.0075620.s002.tiff]

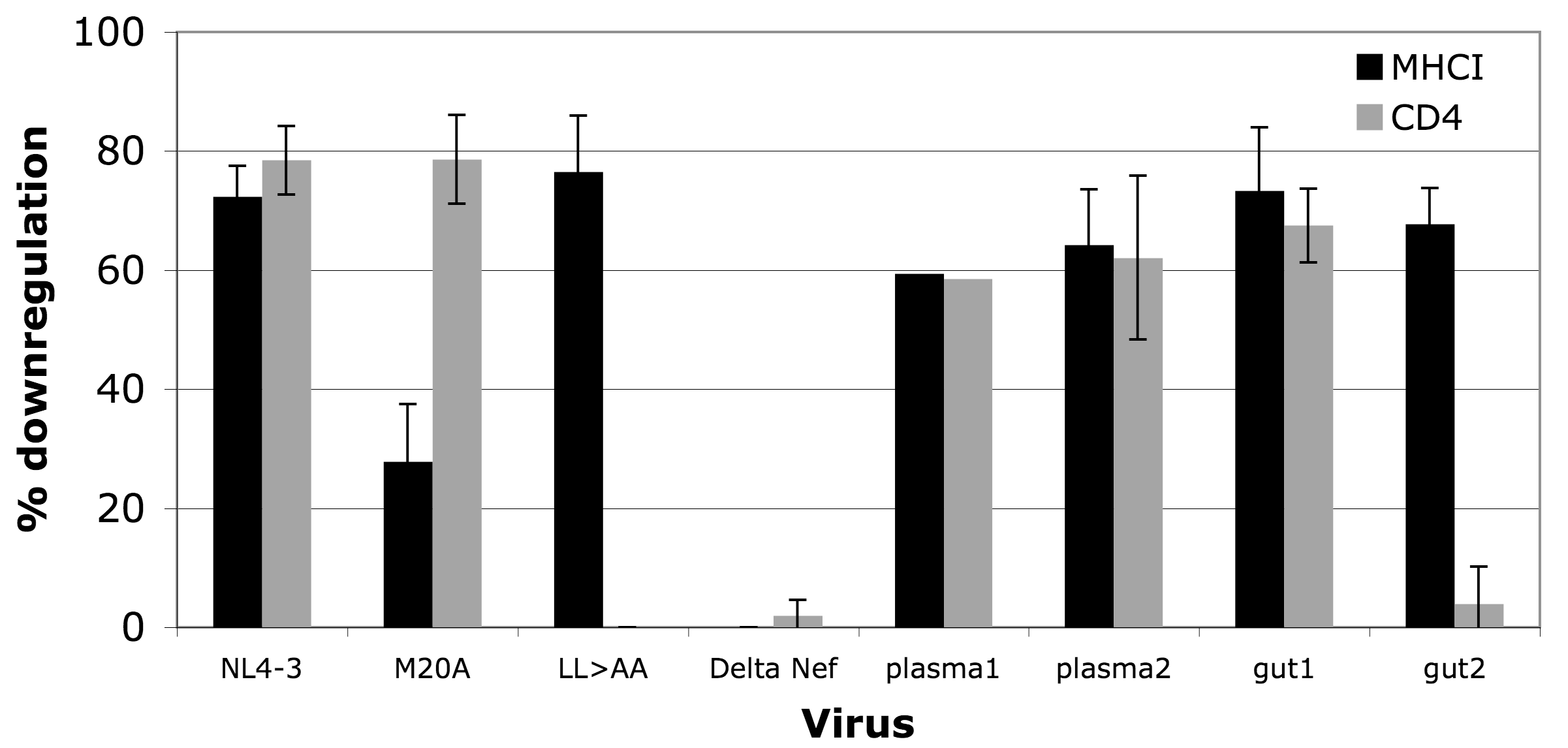

Supplement: Figure S3 — Downregulation by individual clones from subject 650 indicates two different functional populations in the gut. Unfortunately preservation of quasispecies diversity during cloning and virus production could not be confirmed for subject 650. Therefore individual clones were tested for the ability to downregulate MHC Class I (black bars) and CD4 (grey bars). Levels of of HLA-A*02 and CD4 were measured by flow cytometry 3 days after infection of CEM T1 cells with viruses encoding individual plasma- or gut-derived Nef (plasma 1 and 2, gut 1 and 2) or control viruses carrying NL4-3 Nef, Nef LL>AA specifically deficient in CD4 downregulation, and Nef M20A specifically deficient in MHC Class I downregulation. Percent downregulation was determined using Delta Nef and isotype controls. Shown are the average results of 3 separate infections. (TIFF) [file pone.0075620.s003.tiff]

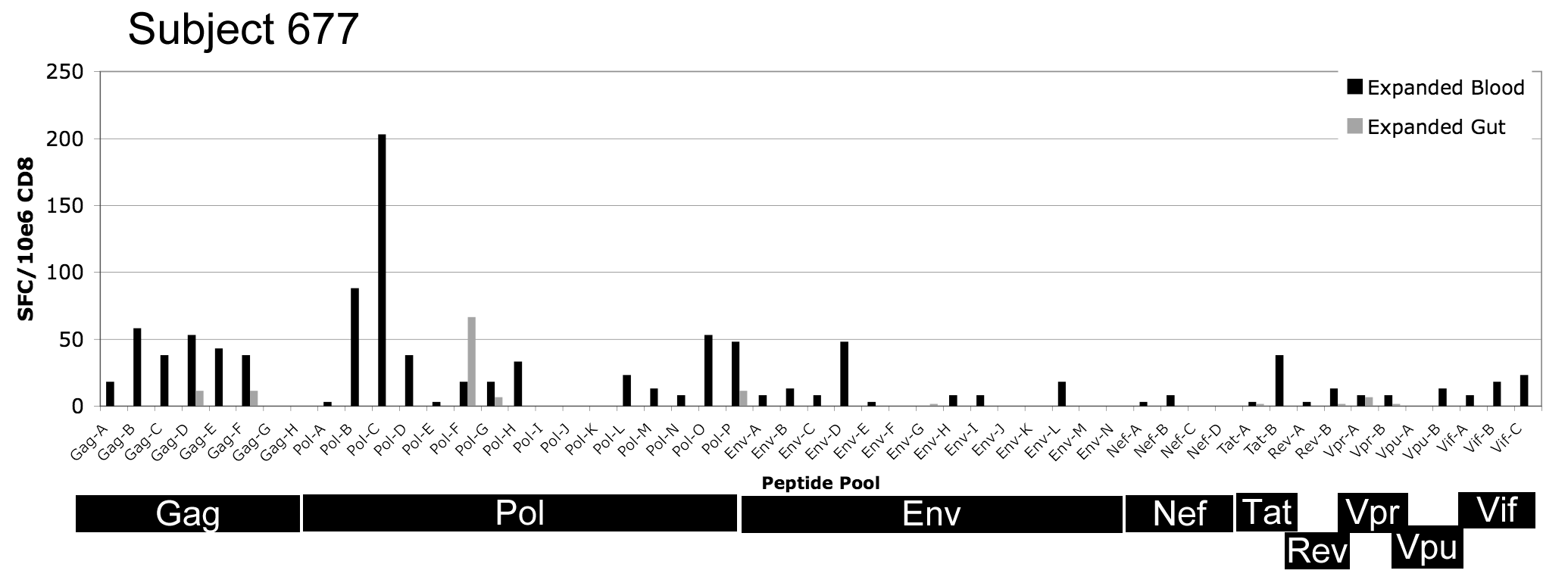

Supplement: Figure S4 — Subject 677 has discordant CTL responses in PBMC vs. MMC. HIV-specific CTL responses were mapped by IFN-gamma ELISpot using expanded CD8+ lymphocytes and pools of Clade B consensus peptides covering the entire HIV proteome. CTL mapping was performed twice with blood and gut biopsy specimens collected on separate visits about 2 weeks apart. ELISpot results showing the mean number of Spot Forming Cells (SFC) per million CD8+ cells for each of the 53 peptide pools. Subject 677 had significantly more and higher magnitude responses from PBMC compared to MMC. (TIF) [file pone.0075620.s004.tif]

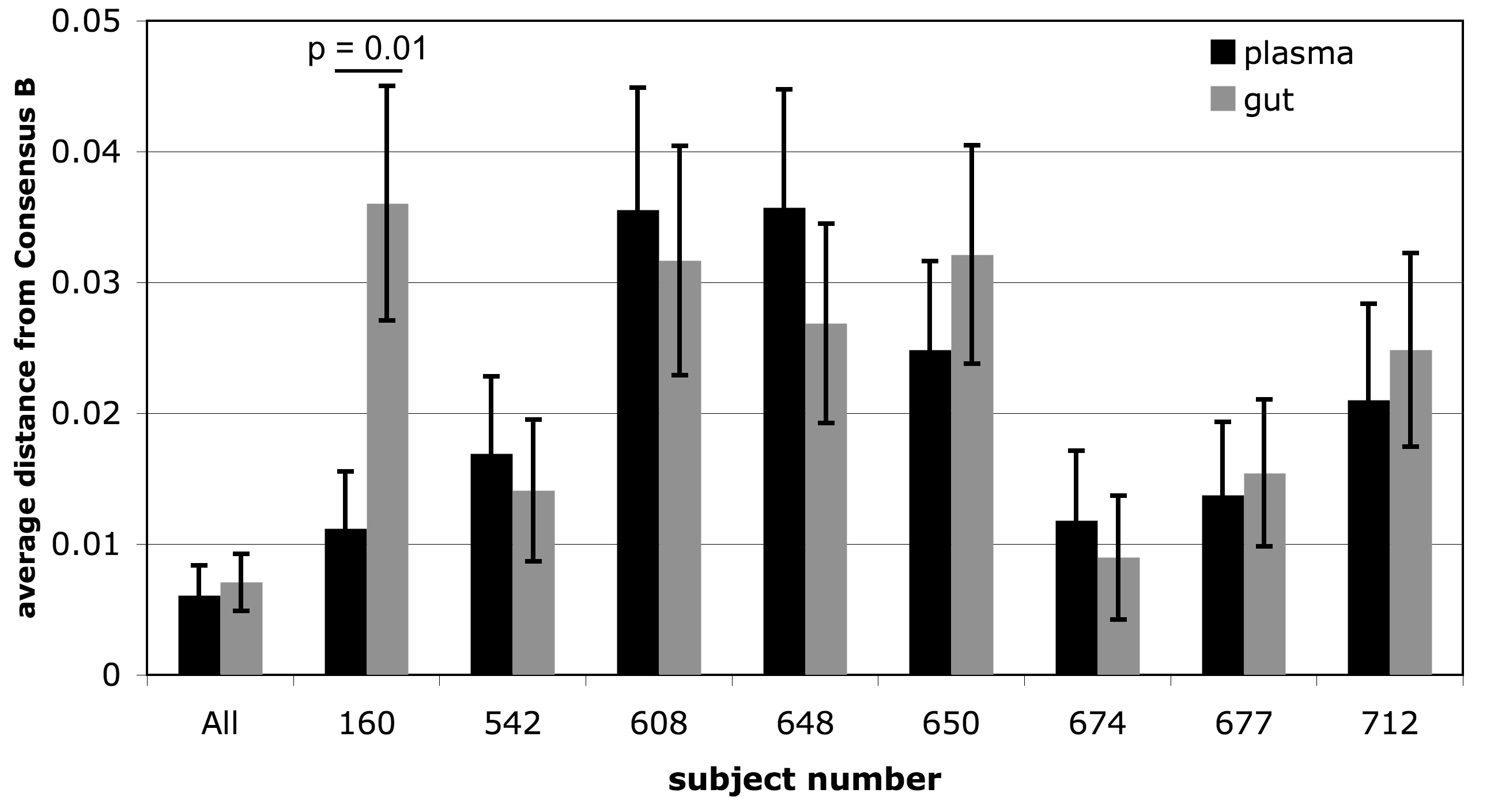

Supplement: Figure S5 — Only subject 160 had a significant difference in divergence from Consensus B between plasma and gut sequences. The divergence of plasma and gut sequences from Consensus B was calculated along with a s.e.m. from 500 bootstrap replicates. The difference in divergence between plasma and gut sequences was evaluated with a two-tailed t test and pairs with significant differences are marked with a bar with the p-value indicated above it. (TIF) [file pone.0075620.s005.tif]

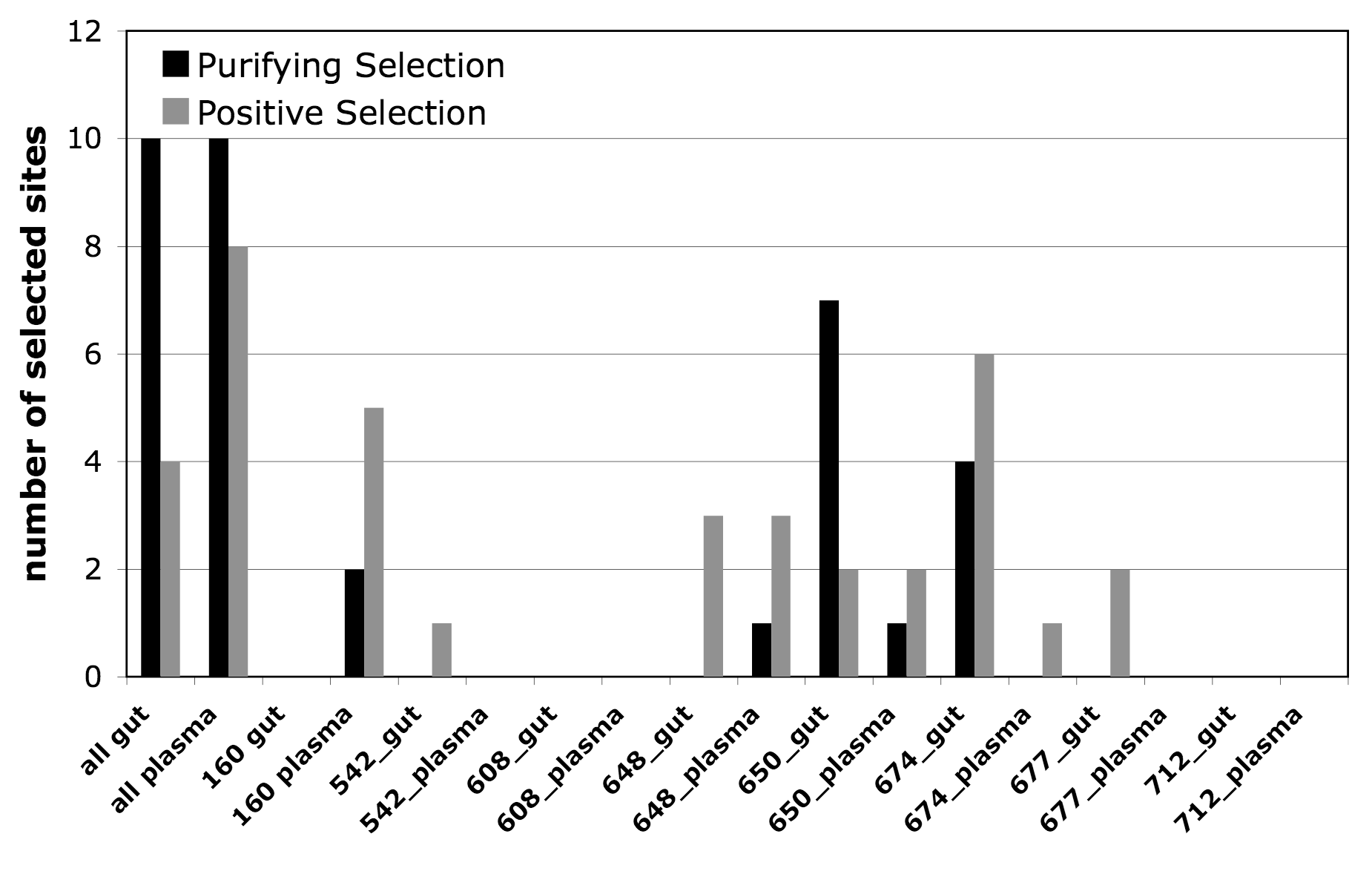

Supplement: Figure S6 — Number of individually selected sites in plasma and gut sequences. Individual amino acid positions with evidence of adaptive evolution were identified by three separate methods, and a site was considered to be adapting under differential selective pressure if that site was identified by at least 2 of 3 methods with a significance level of at least 95% and was only identified in one of the two compartments (gut or plasma). Additionally, only those sites with a dN/dS significantly > and < 1 were considered positive. Subject 650 gut sequences had the highest number of site under purifying selection, while several samples (160gut, 542 plasma, 608 gut and plasma, 712 gut and plasma) had no sites undergoing significant adaptive evoltion. (TIF) [file pone.0075620.s006.tif]
